# Unexpected Diversity of Cellular Immune Responses against Nef and Vif in HIV-1-Infected Patients Who Spontaneously Control Viral Replication

**DOI:** 10.1371/journal.pone.0011436

**Published:** 2010-07-02

**Authors:** Leandro F. Tarosso, Mariana M. Sauer, Sabri Sanabani, Maria Teresa Giret, Helena I. Tomiyama, John Sidney, Shari M. Piaskowski, Ricardo S. Diaz, Ester C. Sabino, Alessandro Sette, Jorge Kalil-Filho, David I. Watkins, Esper G. Kallas

**Affiliations:** 1 Clinical Immunology and Allergy Division, University of Sao Paulo, Sao Paulo, Brazil; 2 Division of Infectious Diseases, Federal University of Sao Paulo, Sao Paulo, Brazil; 3 Sao Paulo Blood Bank, Sao Paulo, Brazil; 4 La Jolla Institute for Allergy and Immunology, La Jolla, California, United States of America; 5 Department of Pathology, Medical School, University of Wisconsin-Madison, Madison, Wisconsin, United States of America; University of Toronto, Canada

## Abstract

**Background:**

HIV-1-infected individuals who spontaneously control viral replication represent an example of successful containment of the AIDS virus. Understanding the anti-viral immune responses in these individuals may help in vaccine design. However, immune responses against HIV-1 are normally analyzed using HIV-1 consensus B 15-mers that overlap by 11 amino acids. Unfortunately, this method may underestimate the real breadth of the cellular immune responses against the autologous sequence of the infecting virus.

**Methodology and Principal Findings:**

Here we compared cellular immune responses against *nef* and *vif*-encoded consensus B 15-mer peptides to responses against HLA class I-predicted minimal optimal epitopes from consensus B and autologous sequences in six patients who have controlled HIV-1 replication. Interestingly, our analysis revealed that three of our patients had broader cellular immune responses against HLA class I-predicted minimal optimal epitopes from either autologous viruses or from the HIV-1 consensus B sequence, when compared to responses against the 15-mer HIV-1 type B consensus peptides.

**Conclusion and Significance:**

This suggests that the cellular immune responses against HIV-1 in controller patients may be broader than we had previously anticipated.

## Introduction

The majority of HIV-1-infected patients progresses to AIDS within 10 to 15 years of infection if they are not treated with antiretroviral drugs. Interestingly, a rare group of individuals remains asymptomatic and has plasma viral loads at low or even undetectable levels. They also maintain high CD4+ T cell counts [1], [2], [3], [4]. The complete understanding of why they are able to control viral replication remains elusive. Several studies have demonstrated that some individuals have been infected by replication-incompetent viruses, which have unusual polymorphisms in *nef* [5], [6], *vpr* [7], and other genes [8]. Vigorous CD4+ [9] and CD8+ T cell [10] responses against the virus have also been detected in some controllers. These immune responses are thought to be pivotal in the beneficial outcome and may be actively containing viral replication [11], [12].

Two early studies have suggested that the HIV-1-specific cytotoxic T lymphocyte (CTL)-mediated response is a major component associated with the control of replication following primary infection [13], [14]. These studies have shown that reduction in HIV-1 viremia during acute infection is associated with the appearance of HIV-1-specific CD8+ T lymphocytes and that the absence of such responses is associated with prolonged symptoms, persistent viremia and antigenemia, and a low CD4+ T cell count. Some HLA alleles, including HLA-B*57 and HLA-B*27, are consistently overrepresented in controllers [15], [16], further suggesting an important role of HLA class-I-restricted CD8+ T cells in these individuals [17].

To characterize CD8+ T cell responses in controllers, peptide scans with HIV-1 consensus B overlapping 15-mer peptides encompassing the viral proteome have been performed [18], [19], [20]. Unfortunately, this method may underestimate the real breadth of the cellular immune responses against the autologous sequence of the infecting virus, since CD8+ T cells recognize epitopes of eight to 11 amino acids in length and the infecting virus sequence is usually significantly different from consensus sequences.

Vif- and Nef-specific CTLs were shown to emerge in elite controller SIV-infected rhesus monkeys after CD8 antibody-mediated depletion and to be actively involved in the control of SIV_mac_239 replication [21]. There is extensive amino acid conservation in the Vif sequences, suggesting functional constraints. Indeed, an HIV-1 mutagenesis study demonstrated that Vif mutants exhibited 25% reduction in a single round of infectivity [22]. Furthermore, Vif is enriched for otherwise rare tryptophans, which often are primary binding anchor residues of several MHC class I molecules, including those associated with slow progression to AIDS [15], [23], [24]. Therefore, boosting CTL responses against Vif and Nef may be an especially potent mechanism of viral suppression and these proteins may have epitopes important for vaccine-induced immunity. Peptide scans of CD8+ T cell-mediated immune responses throughout the Vif consensus B sequence have already been performed identifying 18 epitopes, restricted by five HLA-A and nine HLA-B molecules [25].

Here, we compared cellular immune responses against *vif* and *nef*-encoded consensus B 15-mer peptides to responses against HLA class I-predicted minimal optimal epitopes from consensus B and autologous sequences in six patients who have controlled HIV-1 replication without undergoing antiretroviral therapy. Interestingly, this analysis revealed that three of our patients had broader cellular immune responses against the minimal optimal epitopes than against the 15-mer HIV-1 type B consensus peptide set in ELISPOT-IFN-γ assays. This peptide predicting approach was more sensitive than conventional assays using consensus B peptides and suggests that the cellular immune responses against HIV-1 epitopes may be broader than had been previously anticipated.

## Methods

### Ethics Statement

Informed written consent was obtained from all the patients and the study was approved by the Ethics Committees of the Federal University of Sao Paulo [# 0041/08], University of Sao Paulo [# 4537], and by the Comissão Nacional de Ética em Pesquisa [# 14781].

### Subjects

Blood samples were collected from six male subjects participating in our cohort of recently HIV-1 infected patients [26]. Patients were considered as recently HIV-1-infected when at least one positive HIV-1 ELISA test was confirmed with a Western Blot assay, but was negative using the less sensitive HIV-1 ELISA test Vironostika HIV-1 micro-ELISA system, BioMérieux, Durham, NC. The six patients were classified as controllers of viral replication since most of their plasma HIV-1 RNA loads were below 1,000 copies/mL without antiretroviral therapy.

### CD4+ T cell counts and Viral Load (VL) quantification

Peripheral blood absolute CD4+ T cell counts were assessed using the BD Tritest anti-CD4-FITC/anti-CD8-PE/anti-CD3-PerCP monoclonal antibody (mAb) cocktail and BD TruCount Tubes (BD Biosciences, San Diego, CA), according to the manufacturer's instruction, using a FACSCalibur flow cytometer (BD Biosciences). Plasma HIV-1-RNA was quantified by a standardized reverse transcriptase PCR assay (Amplicor HIV-1 Monitor; Roche Diagnostic Systems, Indianapolis, IN) until January 2007, and subsequently replaced by the branched DNA assay (Versant® - bDNA HIV-1 RNA 3.0 ASSAY, Bayer Health Care LLC Tarrytown, NY).

### HLA class I typing

Subjects were typed with intermediate resolution for major histocompatibility complex (MHC) class I antigen expression using sequence-specific primer PCR kits (Pel-Freez SSP UniTray; Invitrogen, Carlsbad, CA) according to the manufacturer's instructions.

### HIV-1 *vif* and *nef* sequencing

The full-length HIV-1 proviral genome was amplified by nested-PCR in five overlapping fragments of 1.8 to 3.0 kb each and sequenced on an automated sequencer (ABI 3130; Applied Biosystems Inc., Foster City, CA) as previously described [27]. The data from sequenced fragments were edited and assembled into contiguous sequence by the Sequencher program 4.0 (Gene Code Corp., Ann Arbor, MI). The *vif* and *nef* sequences were aligned with the references using the ClustalX 1.81 program, and further hand edited [28]. Phylogenetic trees were created by the maximum likelihood (ML) methods implemented in the program PHYML [29] using the GTR+I+G substitution model and a BIONJ starting tree. A heuristic search for likelihood was performed using the SPR branch-swapping algorithm. Divergence of amino acid substitutions per site within the Vif and Nef sequences was calculated using the Poisson correction method in Mega version 4.0 software.

### HLA class I-restricted epitope prediction and synthesis

We utilized a consensus prediction approach [30], based on the use of several available prediction methods including ARB (average relative binding), SMM (stabilized matrix method) and ANN (artificial neural network) algorithms available through the IEDB (Immune Epitope Database). We also utilized some matrix methods available at NetMHC, and our own PSCL (positional scanning combinatorial library) matrices. ARB, SMM, and ANN predict the quantitative binding affinity. The ANN is a nonlinear model, and ARB and SMM generate scoring matrices [31], [32]. The HIV-1 Vif and Nef consensus B and the autologous amino acid sequences were scanned to identify potential HLA class I binding peptides between nine and 10 residues in length. All possible peptides were scored and ranked for each allele in the donor population using corresponding algorithms. When more than one method was available for a specific allele, the peptides were ranked on the basis of the median score. When an algorithm was not available for a specific allele, predictions were based on an algorithm for another allele in the same allele family, or an allele within the same HLA super type. Candidate peptide sequences ranked in the top 2.5% for each allele were chosen for synthesis. The peptides were then synthesized as crude material. Lyophilized material was resuspended at 20 mg/mL in 100% dimethyl sulfoxide (DMSO) and then diluted to required concentrations in 100% DMSO.

### Interferon gamma (IFN-γ) enzyme-linked immunospot (ELISPOT) assay

Two sets of ELISPOT assays were carried out. In the first set, 15-mer peptides overlapping by 11 amino acids corresponding to HIV-1 consensus B Vif and Nef (NIH AIDS Research and Reagent Program, Rockville, MD) were used at a final concentration of 10 µg/mL. In the second set of ELISPOT assays, HLA class I-restricted Vif and Nef peptides of nine or 10 amino acids in length (predicted as described above) were used at the same concentration. For each patient, the number of HLA-restricted peptides tested ranged from 70 to 110. Out of these peptides, some were consensus B-based and others were autologous sequences-based (See supporting information Table S1: Number of peptides tested for each patient in the ELISPOT assays). Briefly, 96-well flat-bottomed nitrocellulose plates (Multiscreen, Millipore, Bedford, MA) were coated with 0.5 µg of anti-IFN-γ mAb (Mabtech, Nacka, Sweden) for a 1 h incubation at 4°C. Plates were washed three times with 1xPBS and then 1×10^5^ PBMC resuspended in RPMI 1640 supplemented with penicillin, streptomycin, and 10% fetal bovine serum (R10) were added to the wells. The R10 also contained each Vif or Nef peptide, Concanavalin-A (positive control), or only 2.0 µL of 100% DMSO with no peptide (negative control). Plates were incubated for 16 h at 37°C in 5% CO_2_, after which the cells were discarded. After washing the plates five times with 1xPBST (1xPBS containing 0.05% Tween 20), 0.05 µg of biotinylated anti-IFN-γ mAb (Mabtech) was added and the plates were incubated for further 2 h at room temperature. Following additional five washes with 1xPBST, 0.07 µg per well of alkaline phosphatase (Vector Laboratories Inc., Burlingane, CA) in 50 µL of 1xPBS were added for 1 h at room temperature. After another set of five washes with 1xPBST, 50 µL of BCIP/NBT (5-Bromo-4-Chloro-3-Indolyl Phosphate/Nitro Blue Tetrazolium) substrate solution (Sigma-Aldrich, St Louis, MO) per well were added, and the plates were developed for about 30 min. Spots were counted using either an automated stereomicroscope (KS ELISPOT, Zeiss, Oberkochem, Germany) or AID reader (AID, Germany). HIV-1-specific responses were reported as the number of spot-forming units (SFU)/1×10^6^ PBMC after subtraction of the background IFN-γ secretion. A response was considered positive if the number of SFU exceeded 55 SFU/1×10^6^ cells and was at least four-times the level of the wells with no peptide [33] (See supporting information Figure S1: ELISPOT assays using 15-mer consensus B peptides and HLA-restricted minimal optimal peptides).

## Results

In the present study, we examined cellular immune responses in six HIV-1-infected subjects who controlled viral replication. All of them had documented infection for at least three years, with a mean duration of four years. We used ELISPOT-IFN-γ assays with 15-mers overlapping by 11 amino acids encompassing the entire length of the Vif and Nef consensus B sequences and HLA class I-restricted minimal optimal Vif- and Nef-epitopes synthesized from consensus B and autologous virus sequences.

### CD4+ T cell counts and VL quantification

The six controller patients were infected through unprotected homosexual activity and have been followed-up since diagnosis in the early stage of the infection. HIV-1 RNA levels in the plasma and peripheral CD4+ T cell counts were determined approximately every three months for at least 1,200 days. The majority of VL quantifications was below 1,000 copies/mL for all the patients and the mean CD4+ T cell count was 642 cells/µL varying from 353 to 1097 cells/µL (Table 1). Only two individuals (1068 and 1022) had occasional viral loads above 2,000 copies/mL during their more recent follow-up. Patient 1098 had occasional viral loads above 1,000 copies/mL. Patients 1073, 1103, and 2017 presented all viral loads below the detection limits (Supporting information Figure S2: Viral load in the controller patients). No patient was homozygous for the *CCR5*Δ32 polymorphism, and only patient 1068 was heterozygous for this deletion. We also looked for active GBV-C infection in our patients, and none of them tested positive for virus RNA.

**Table 1:** HLA type and clinical characteristics of study patients.

Patient ID	HLA				Highest VL (copies/mL)	CD4+ T cell count range (cells/µL)	Follow-up (days)
	A locus alleles		B locus alleles				
**1022**	02	02	14	52	2,090	384–819	2063
**1068**	03	29	70	58	7,260	721–1035	1546
**1073**	01	34	35	57	<400	433–762	1498
**1098**	03	26	27	57	807	654–882	1169
**1103**	01	02	35	57	<400	601–909	1162
**2017**	02	11	39	53	<400	518–1030	1904

### The HLA-B*57 allele was overrepresented in subjects who controlled viral replication

We typed 157 individuals from our cohort of HIV-1 recently infected patients for their HLA class I alleles. Among the six controllers, three out of them were positive for the HLA-B*57 allele (Table 1), which has been associated with restriction of HIV-1 replication [1], [15]. The overall frequency of individuals positive for the HLA-B*57 allele in the entire cohort was 5% (eight out of 157 patients).

### Characterization of the HIV-1 genome and genetic diversity

**Figure 1:**
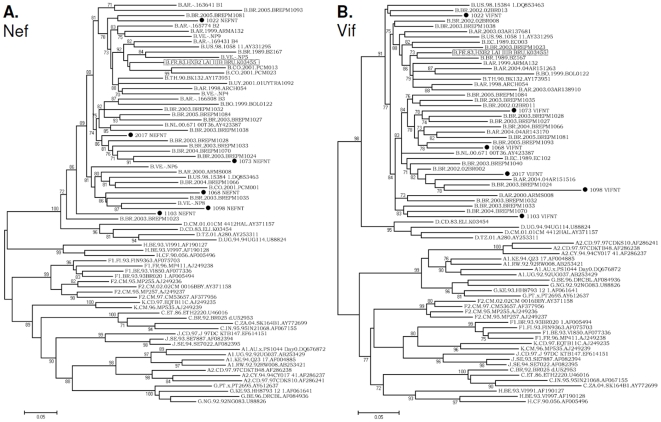
Maximum likelihood phylogenetic trees. Trees of *nef* (**A**) and *vif* (**B**) sequences from six controller patients (black circles) along with references for all known subtypes from the LANL database (labeled branches) are displayed. HXB2_LAI_IIIB_BRU (K03455) HXB2 reference sequence is boxed. The construction of the trees is described in the text. For purposes of clarity, the trees were midpoint rooted. The aLRT values of ≥70% are indicated at nodes. The scale bars represent 0.05 nucleotide substitution per site. Clustering of the six controllers with subtype B is evident in *nef* and *vif* trees (aLRT ≥85%).

Phylogenetic analysis of the nucleotide sequences of vif and nef regions showed that all six controller patients were infected with subtype B strains. The segregation patterns of *vif* and nef sequences were randomly distributed among subtype B reference sequences and other non-controller patients' sequences without apparent clustering (Figure 1). All of the controller patients had an intact genomic organization with open reading frames. No gross insertion or deletion was observed in both *vif* and *nef* regions. In our six controller patients, the HIV-1 *vif* and *nef* autologous sequences differed from the HIV-1 consensus B sequences by an average value of 15.1% and 13.6%, respectively. The overall mean amino acid distances of the six controllers' sequences compared to consensus B sequences from the reported positive Vif and Nef epitopes were 19.4% (range; 9.7%–28.3%), and 18.1% (range; 10.8%–25.4%), respectively.

### HLA-class I-restricted epitope prediction

CD8+ T cells preferentially recognize MHC class I-bound peptides of eight to 11 amino acids in length. T cell responses against consensus B 15-mer peptides might, therefore, not fully capture the complexity of T cell recognition of epitopes *in vivo*. To address this issue, we scanned full-length amino acid sequences of the Vif and Nef proteins predicted from autologous viruses. For a comparison sake, we also scanned consensus B peptides using the same HLA class I algorithms. We identified 558 optimal, HLA-restricted 9- or 10-mer peptides (70 to 110 per patient) that were predicted to bind to the HLA-class I A and B molecules of the patients (Supporting information Table S1). After synthesis, these 558 peptides were used individually in ELISPOT-IFN-γ assays.

### Cellular immune responses detected against 15-mer consensus B peptides

**Figure 2:**
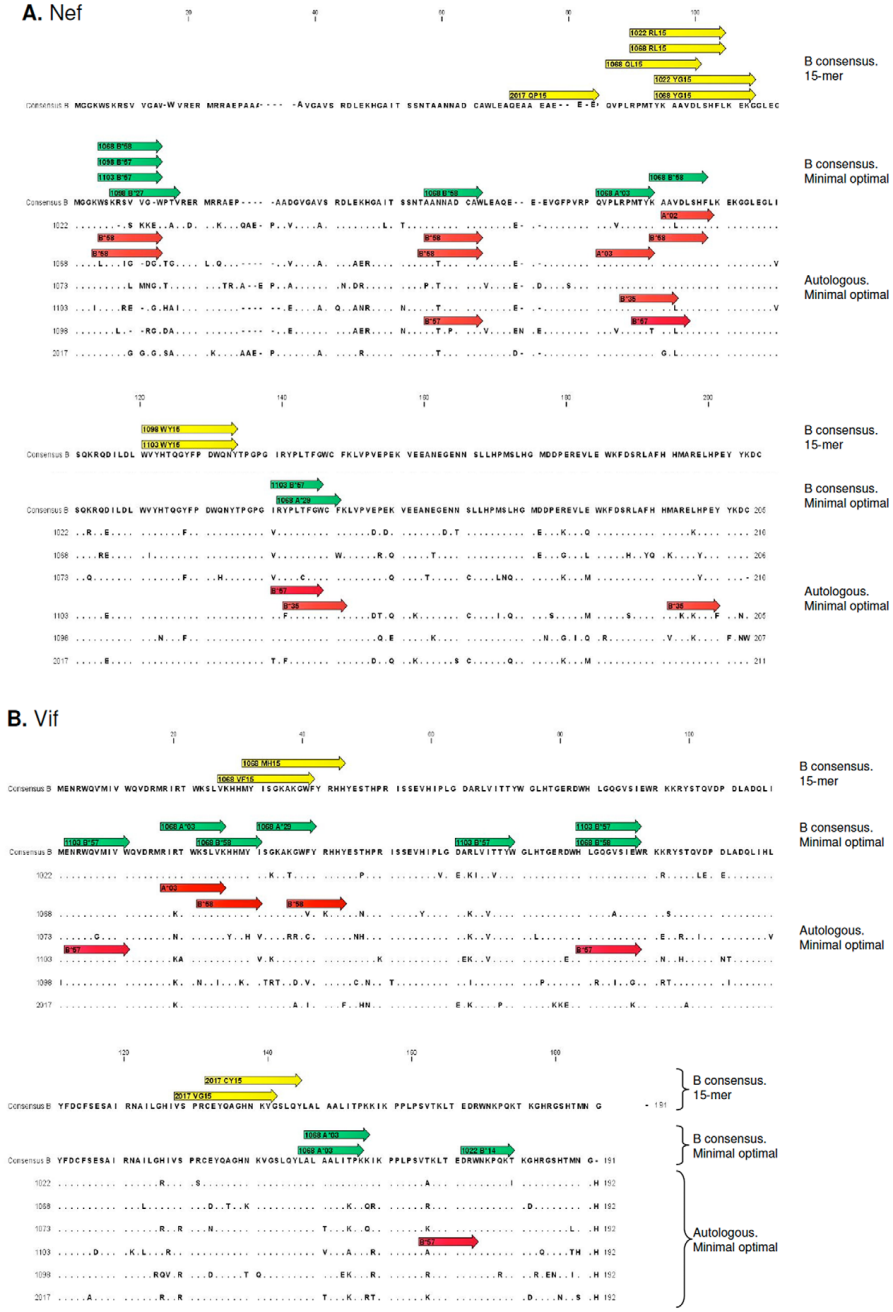
Alignments of the viral sequences and maps of the cellular immune responses. Alignments and maps of immune response from six HIV controller patients to consensus B sequence and maps of the cellular immune responses detected in the ELISPOT assays. The arrows represent the peptides, which elicited positive IFN-γ responses. Yellow arrows show responses against consensus B 15-mers, green arrows show responses against consensus B minimal optimal HLA-restricted peptides, and red arrows show responses against autologous sequences minimal optimal HLA-restricted peptides. **A**. First sequence is the map of immune response against consensus B 15-mer peptides for Nef. Other sequences show the maps of immune responses detected against minimal optimal epitopes from consensus B and autologous sequences. **B**. Vif sequences, see Fig. 2A legend for explanation.

Five of our six patients recognized Nef epitopes and two of them recognized Vif epitopes using ELISPOT assays with the consensus B 15-mer peptides. Of the 49 peptides that span the Nef sequence (205 amino acids in length), five were reactive. Four 15-mers of the 46 spanning the entire Vif sequence (191 amino acids in length) induced significant production of IFN-γ in the ELISPOT assays (Supporting information Table S2, Figure 2). The region between amino acids 61 and 95 of the Nef consensus B sequence contained more T cell epitopes than any other region; six of the eight positive responses were located in this region. However, similar epitope concentrations were not observed in Vif (Supporting information Table S2, Figure 2).

### HLA class I-restricted minimal optimal epitopes revealed a broader immune response than 15-mer consensus B peptides

T cells from three controller patients recognized additional epitopes when ELISPOT assays were carried out with the minimal optimal epitopes, compared to the assays using the consensus B 15-mers. For the Nef HLA class I-restricted epitopes, 22 positive responses were detected (Supporting information Table S2, Figure 2A). For the Vif HLA class I-restricted peptides, there were 16 IFN-γ responses (Supporting information Table S2, Figure 2B). We found positive responses to peptides restricted to eight HLA class I molecules. The majority of these responses was restricted by *B* locus alleles (74%) and was directed against Nef epitopes (63%).

Like the 15-mer consensus B peptides spanning Nef, T cells recognized minimal optimal epitopes concentrated in the area between amino acids 61 and 95. In this region, we detected seven of the 22 responses found. Interestingly, we observed an additional concentration of T cell responses against minimal optimals in the first 20 amino acids of Nef (Figure 2A). Six positive responses were detected in this region. By contrast, analyses of the HLA-restricted responses against Vif did not reveal any epitope rich region (Figure 2B).

We identified three regions (two in Nef and one in Vif) where 15-mers peptides (Figure 2, yellow arrows) elicited positive responses not seen after minimal optimal peptides stimulation (Figure 2, green and red arrows). Specifically, the Nef WY15 peptide was recognized by patients 1103 and 1098 and the QP15 peptide by patient 2017. The Vif VG15 peptide and the CY15 peptide were recognized by patient 2017. No minimal optimal peptides were predicted in these regions by the employed bioinformatics tools using the autologous and consensus B sequences. Recognition of these 15-mers was a relatively rare event when compared to the large number of HLA-predicted epitopes that were recognized.

**Figure 3:**
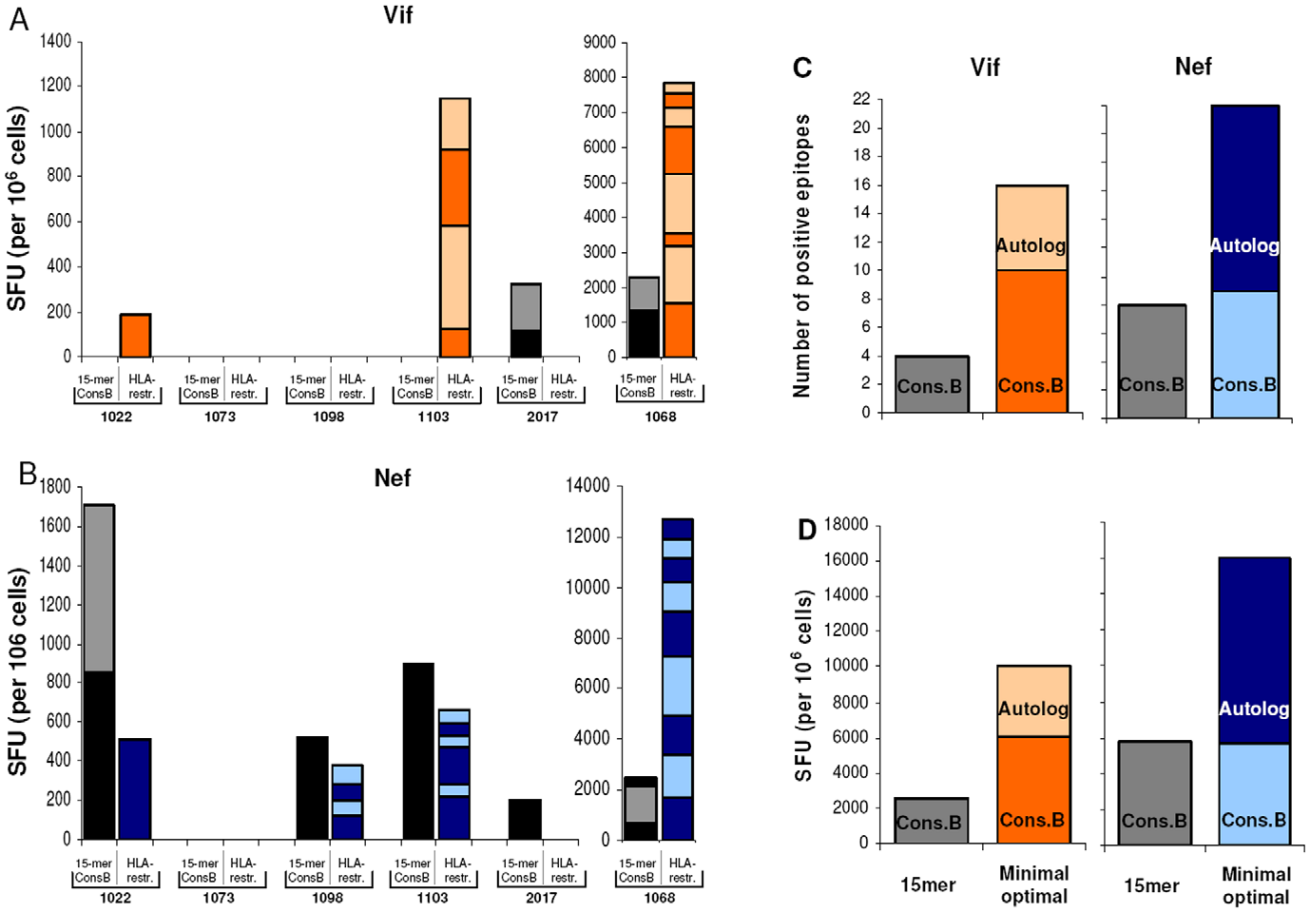
Cellular immune responses against consensus B-based and autologous-based Vif and Nef peptides. Stacked bars show results of the ELISPOT assays for each of the six controller patients for Vif (**A**) and Nef (**B**). Each portion of the bar indicates one peptide and its height represents the amount of spots shown in the assays. The left black/gray bar for each patient shows positive spots detected with 15-mer peptides based on the consensus B sequence (15-mer/ConsB) and right colored bar shows positive spots detected with minimal optimal HLA-restricted peptides (HLA-restr.). **C**. Reactivity of PBMC in the ELISPOT assays to consensus B 15-mers and HLA-restricted minimal optimal epitopes from consensus B and autologous sequences in terms of number of recognized epitopes. **D**. The magnitude of the response in spot forming units per 10^6^ cells.

We detected more T cell responses against Vif and Nef when we used HLA class I-restricted minimal optimal epitopes than when we used the conventional approach of testing consensus B 15-mers (Figure 3A and 3B, respectively). Indeed two aspects of T cell recognition were shown to be higher using this approach: the number of epitopes eliciting positive responses and the total number of SFU detected in the assays (Figure 3C and 3D, respectively).

We also compared the ability of PBMC from our six patients to recognize consensus B 15-mers, HLA-restricted minimal optimal epitopes in the consensus B sequence and HLA-restricted minimal optimal epitopes in the autologous HIV-1 sequences. Reactivity was higher (in terms of frequency and the number of epitopes recognized) using the HLA-restricted minimal optimal epitopes in the consensus B and autologous virus. Indeed, when we used either consensus B or autologous sequence to synthesize HLA-restricted minimal optimal epitopes, a higher number of positive responses were identified. For Vif, three out of the six controllers had increased positive responses against HLA-restricted minimal optimals while two patients remained without detectable responses (Figure 3A). For Nef, three out of the six patients had a higher number of positive responses against HLA-restricted peptides, and one patient remained without detectable responses (Figure 3B). Interestingly, the consensus B HLA-restricted minimal optimal epitopes proved to be more sensitive in the detection of Vif-specific responses, whereas the autologous HLA-restricted minimal optimal epitopes were more sensitive for the detection of Nef-specific responses (Supporting Information Table S2, Figure 3C and 3D).

## Discussion

HIV-1 controllers, although rare, represent about 1% of HIV-1-infected individuals [34]. The controller patients studied here have been monitored for at least three years since early infection. Overall, they have had CD4+ T cell counts (mean number during all follow-up visits of 642 cells/mm^3^) and plasma viral loads (<2,000 copies/mL) similar to other previously reported cohorts of controller patients [3], [34], [35]. In this study, we demonstrated that assessing T cell responses in subjects who successfully control viral replication with consensus B 15-mer peptides may be less sensitive when compared to HLA-restricted minimal optimal epitopes. Indeed, by HLA-restricted minimal optimals, we have discovered broad Vif and Nef-specific T cell responses in our patients.

As viral load relates to transmission and disease progression [36], [37], further comprehension of the mechanisms by which some infected individuals spontaneously control HIV-1 replication could assist in the development of vaccines that augment control of infection, reduce risk of transmission, and ameliorate disease progression [35], [38]. It is, therefore, critical to clearly define protective cellular immune responses in HIV-1-infected subjects who successfully control viral replication and then to engender these responses by vaccination.

Some HLA class I alleles can influence disease outcome, including control of viremia [3], [15], [23], [34]. Overrepresentation of HLA-B*57 (approx. 85%) was found in HIV-1 controllers in several studies [1], [3], [15]. Not surprisingly, we found the same allele enrichment in our controller patients since three out of the six patients expressed HLA-B*57. In our entire cohort of recently HIV-1 infected individuals, the frequency of individuals carrying this allele was less than 5%. Despite the strong association with viral control, it is unclear whether HLA-B*57 mediates its effects entirely via HIV-1-specific CD8+ T cell responses or by other mechanisms. It has been reported that carriers of this allele often mount a potent T cell response against one or more highly conserved viral epitopes, but viral escape from these responses is not always associated with viral rebound [39]. Also, it is well known that some individuals control viral replication without carrying “protective” HLA alleles [1].

An auxiliary gene of primate immunodeficiency viruses, *vif* is required for HIV-1 replication in lymphocytes and macrophages [40], [41], [42] and is consequently maintained intact *in vivo* in infected people [43]. Vif was recently found to counteract the host antiviral factors APOBEC3G and APOBEC3F by binding to them and promoting their degradation. Hassaïne *et al.* [44] found that the precise amino acid signature Ser132Arg in Vif correlated with a five-fold reduction in replication in long term nonprogressor. Interestingly, sequencing of the *vif* gene showed that only patient 1022 had a virus carrying this mutation (Figure 2B). By contrast, the very conserved ^144^SLAQXLA^149^ residue, known to be one of the binding sites for APOBEC [45] was invariant in the six controllers described here.

The importance of Nef in the pathogenesis of HIV-1 was investigated after transmission of the virus from an HIV-1-infected blood donor to six recipients. Their CD4+ T cell counts have been stable and normal for 10 to 14 years after transfusion. Their virus sequences showed similar deletions in the *nef* gene. In our study, we found no marker in the *nef* sequence that could explain the transmission of an attenuated virus to our patients. Phylogenetic analysis of *nef* and *vif* sequences (Figures 1A and 1B, respectively) revealed that the viral genes from the controller subjects inter digitated with sequences from other recently HIV-1-infected non-controller individuals, without apparent clustering. Our observations agree with some results from a single codon-level comparison of all coding HIV-1 genes among 95 elite controllers and persons with progressive infection. This study indicated that the ability to spontaneously control of HIV-1 replication was not attributed to either specific viral genetic polymorphism or gross viral genetic defects [46].

Natural history studies in cohorts of HIV-1-infected humans and analogous SIV studies in macaques showed that cell-mediated immunity can control primate immunodeficiency virus replication [34]. Friedrich et al. [21] examined Indian-origin rhesus macaques presenting robust and durable control of SIV_mac_239 viremia during chronic infection. By depleting CD8+ lymphocytes from these animals, they discovered that Nef- and Vif-specific CD8+ T cell responses along with robust Gag-specific CD4+ T cell responses were responsible for the control of the SIV_mac_239 replication. While Nef and Gag have been used in vaccine development, Vif has not been used as a vaccine immunogen to date. Therefore, it is possible that boosting CTL responses against Vif may be an especially potent mechanism of suppressing viral replication.

To determine a method to assess CD8+ T cell responses to HIV-1 by measuring intracellular cytokine production, Betts *et al.* [47] compared responses to HIV-1 Gag 15-mers overlapping by 11 amino acids to responses elicited by optimized epitopes of eight to 11 amino acids in length and observed a marked difference in reactivity. They hypothesized that the overlapping 15-mers panels may underestimate the total HIV-1-specific response. This could be explained by a number of factors such as the location of the recognized sequence within the 15-mer, the effects of amino acid overlaps, and potential sequence differences between peptides and autologous virus. In fact, in our study population, the HIV-1 *vif* and *nef* autologous sequences differ from HIV-1 consensus B sequences by an average value of 15.1% and 13.6%, respectively.

A previous study has evaluated the cellular immune response against other proteins using autologous virus isolates. Altfeld et al. [48] demonstrated the usefulness of autologous sequences to detect immune responses against Tat and Vpr. They also suggested that the entire immune response to the virus may be underestimated using consensus sequence based peptides. However, this effect could not be detected for Gag-specific responses, which could reflect less structural changes in this protein compared to Tat and Vpr.

In our study, some regions in Nef and Vif engendered positive responses when we used 15-mers but not when we used HLA-restricted minimal optimal peptides (e.g. the Nef peptide WY15 and the Vif peptide VG15, Figure 2). Since 15-mers peptides are know to stimulate both CD4+ and CD8+ T cells [47], it is possible that some of these 15-mer-specific responses were mediated by CD4+ T cells or were restricted by HLA class I locus Cw molecules. Thus, 15-mers can detect both CD4- and CD8-mediated T cell responses. Conversely, the HLA-class I restricted minimal optimal epitopes are expected to stimulate only CD8 T cells. One might, therefore, expect more responses against the 15-mers. However, in our study, the HLA-restricted minimal optimal epitopes engendered more responses than the 15-mers did, suggesting that these minimal optimal epitopes are much better at detecting CD8 T cell responses than the corresponding 15-mers. The inability of the employed bioinformatics approach to predict these particular epitopes and the possibility of escape could also explain why some responses were detected with 15-mers but were not detected using autologous HLA-restricted epitopes. We did not, however, investigate whether these epitopes escaped by sequencing the virus at a second time point.

Collectively, our data suggest that Nef and Vif-specific responses may be broader than we had anticipated. HLA-restricted minimal optimal peptides increased of our ability to detect positive cellular responses against Nef and Vif. In conclusion, our study suggests that selection of peptides based on an individual's HLA class I allele, either using autologous or consensus B sequences, may be important in the definition of antigen-specific CD8+ T cell responses. This is likely to be true not only in subjects who successfully control viral replication but in any HIV-1-infected individual.

## Supporting Information

Table S1 Number of peptides tested for each patient in the ELISPOT assays.
(0.04 MB DOC)

Table S2 Positive peptides in the ELISPOT assays. *per 10^6^ cells; **Columns “Source” indicate whether the HLA-restricted peptides were predicted from Consensus B (Cons) or autologous sequences ([Patient ID]). In some cases, the Consensus B and the autologous sequences are identical, so the source of the peptide is indicated as Cons/[Patient ID].
(0.01 MB PDF)

Figure S1 ELISPOT assays using 15mer consensus B peptides and HLA-restricted minimal optimal peptides. ELISPOT assays were done as described in the Methods section using either Vif and Nef 15mer consensus B peptides or Vif and Nef HLA-restricted minimal optimal peptides. For both assays, a concentration of 10 µg/mL for each peptide tested separately was used. A response was considered positive if the number of SFU exceeded 55 SFU per 10^6^ cells and was at least four-times the level of the wells with no peptide.
(4.11 MB TIF)

Figure S2 Viral load in the controller patients. Quantifications of viral load were performed by RT-PCR or branched DNA assays. The graph shows the values using a log scale.
(6.22 MB TIF)
