# Correction: Unexpected Diversity of Cellular Immune Responses against Nef and Vif in HIV-1-Infected Patients Who Spontaneously Control Viral Replication

**DOI:** 10.1371/annotation/3cf88893-7718-4aaa-b518-cf8ed592685e

**Published:** 2013-09-09

**Authors:** Leandro F. Tarosso, Mariana M. Sauer, Sabri Sanabani, Maria Teresa Giret, Helena I. Tomiyama, John Sidney, Shari M. Piaskowski, Ricardo S. Diaz, Ester C. Sabino, Alessandro Sette, Jorge Kalil-Filho, David I. Watkins, Esper G. Kallas

A second grant from the National Institutes of Health to the twelfth author was incorrectly omitted from the funding statement. The number of this grant is: R21 AI073230-01. 

