# Supplementary material for: Unexpected Diversity of Cellular Immune Responses against Nef and Vif in HIV-1-Infected Patients Who Spontaneously Control Viral Replication
Source: PLoS One. 2010 Jul 2;5(7):e11436. doi: 10.1371/journal.pone.0011436 (PMC2896403; doi:10.1371/journal.pone.0011436)
Supplement: Table S1 — Number of peptides tested for each patient in the ELISPOT assays. (0.04 MB DOC) [file pone.0011436.s001.doc]

**Table S1.** Number of peptides tested for each patient in the ELISPOT assays

| **Patient** | **HLA-restricted** | | | **Consensus B  15-mers** |
| --- | --- | --- | --- | --- |
| **ID** | **Total** | **Autologous** | **Consensus B** |  |
| **1022** | 70 | 34 | 36 | 95 |
|  | (Vif: 35; Nef: 35) | (Vif: 16; Nef: 18) | (Vif: 19; Nef: 17) | (Vif: 46; Nef: 49) |
| **1068** | 84 | 35 | 49 | 95 |
|  | (Vif: 38; Nef: 46) | (Vif: 16; Nef: 19) | (Vif: 22; Nef: 27) | (Vif: 46; Nef: 49) |
| **1073** | 100 | 51 | 49 | 95 |
|  | (Vif: 51; Nef: 49) | (Vif: 28; Nef: 23) | (Vif: 23; Nef: 26) | (Vif: 46; Nef: 49) |
| **1098** | 94 | 42 | 52 | 95 |
|  | (Vif: 53; Nef: 41) | (Vif: 27; Nef: 15) | (Vif: 26; Nef: 26) | (Vif: 46; Nef: 49) |
| **1103** | 100 | 44 | 56 | 95 |
|  | (Vif: 47; Nef: 53) | (Vif: 23; Nef: 21) | (Vif: 24; Nef: 32) | (Vif: 46; Nef: 49) |
| **2017** | 110 | 50 | 60 | 95 |
|  | (Vif: 57; Nef: 53) | (Vif: 26; Nef: 24) | (Vif: 31; Nef: 29) | (Vif: 46; Nef: 49) |
