# Supplementary material for: Unexpected Diversity of Cellular Immune Responses against Nef and Vif in HIV-1-Infected Patients Who Spontaneously Control Viral Replication
Source: PLoS One. 2010 Jul 2;5(7):e11436. doi: 10.1371/journal.pone.0011436 (PMC2896403; doi:10.1371/journal.pone.0011436)
Supplement: Table S2 — Positive peptides in the ELISPOT assays. *per 106 cells; **Columns “Source” indicate whether the HLA-restricted peptides were predicted from Consensus B (Cons) or autologous sequences ([Patient ID]). In some cases, the Consensus B and the autologous sequences are identical, so the source of the peptide is indicated as Cons/[Patient ID]. (0.01 MB PDF) [file pone.0011436.s002.pdf]

Table S2. Positive peptides in the ELISPOT assays

Patient ID	15-mer peptides from Consensus B sequence						Minimal optimal peptides from autologous viruses and Consensus B sequences										
	Nef			Vif			Nef					Vif				
	Sequence	Position	SFU*	Sequence	Position	SFU*	Sequence	Position	Source**	HLA restriction	SFU*	Sequence	Position	Source**	HLA restriction	SFU*
**1022**	RPMTYKAAVDLSHFL	Nef: 77-91	850	no response			AALDLSHFL	Nef: 87-95	1022	A*0201	515	DRWNKPQKT	Vif: 172-180	Cons	B*1402	185
	YKAAVDLSHFLKEKG	Nef: 81-95	860													
**1068**	QVPLRPMTYKAAVDL	Nef: 73-87	705	VKHHMYISGKAKGWF	Vif: 25-39	1360	WSKRSVVGW	Nef: 5-13	Cons	B*5801	1560	RIRTWKSLVK	Vif: 17-26	Cons	A*0301	1645
	RPMTYKAAVDLSHFL	Nef: 77-91	1450	MYISGKAKGWFYRHH	Vif: 29-43	925	AANNADCAW	Nef: 49-57	Cons	B*5801	1175	ISGKAKGWFY	Vif: 31-40	Cons	A*2902	1695
	YKAAVDLSHFLKEKG	Nef: 81-95	290				RYPLTFGWCF	Nef: 134-143	Cons	A*2902	760	HLGQGVSIEW	Vif: 80-89	Cons	B*5801	550
							QVPLRPMTYK	Nef: 73-82	Cons/1068	A*0301	965	LALAALITPK	Vif: 148-157	Cons	A*0301	410
							KAAVDLSHF	Nef: 82-90	Cons/1068	B*5801	780	ALAALITPKK	Vif: 149-158	Cons	A*0301	300
							KLSKRIGDGW	Nef: 4-13	1068	B*5801	1665	KSLVKHHMYI	Vif: 22-31	Cons/1068	B*5801	390
							LSKRIGDGW	Nef: 5-13	1068	B*5801	1710	RIKTWKSLVK	Vif: 17-26	1068	A*0301	1535
							TAATNADCAW	Nef: 48-57	1068	B*5801	2320	KGWVYKHHY	Vif: 36-44	1068	B*5801	1330
							AATNADCAW	Nef: 49-57	1068	B*5801	1770					
**1073**	no response			no response			no response					no response				
**1098**	WVYHTQGYFPDWQNY	Nef: 113-12[illegible]	525	no response			WSKRSVVGW	Nef: 5-13	Cons	B*5701	120	no response				
							KRSVVGWPTV	Nef: 7-16	Cons	B*2705	80					
							AATNPDCAW	Nef: 48-56	1098	B*5701	80					
							MTYTAALDL	Nef: 80-88	1098	B*5701	100					
**1103**	WVYHTQGYFPDWQNY	Nef: 113-12[illegible]	895	no response			WSKRSVVGW	Nef: 5-13	Cons	B*5701	220	DARLVITTYW	Vif: 61-70	Cons	B*5701	460
							IRYPLTFGW	Nef: 132-140	Cons	B*5701	60	ENRWQVMIVW	Vif: 2-11	Cons/1103	B*5701	125
							RPMTYKAAL	Nef: 76-84	1103	B*3501	65	HLGQGVSIEW	Vif: 80-89	Cons/1103	B*5701	335
							IRFPLTFGW	Nef: 132-140	1103	B*5701	185	VAKLTEDRW	Vif: 166-174	1103	B*5701	225
							FPLTFGWCF	Nef: 134-142	1103	B*3501	60					
							MAKEKHPEF	Nef: 193-201	1103	B*3501	75					
**2017**	QEEEEVGFPVRPQVP	Nef: 61-75	205	VSPRCEYQAGHNKVG	Vif: 129-143	120	no response					no response				
				CEYQAGHNKVGSLQY	Vif: 133-147	200										
