# Supplementary figures and images for: Unexpected Diversity of Cellular Immune Responses against Nef and Vif in HIV-1-Infected Patients Who Spontaneously Control Viral Replication
Source: PLoS One. 2010 Jul 2;5(7):e11436. doi: 10.1371/journal.pone.0011436 (PMC2896403; doi:10.1371/journal.pone.0011436)

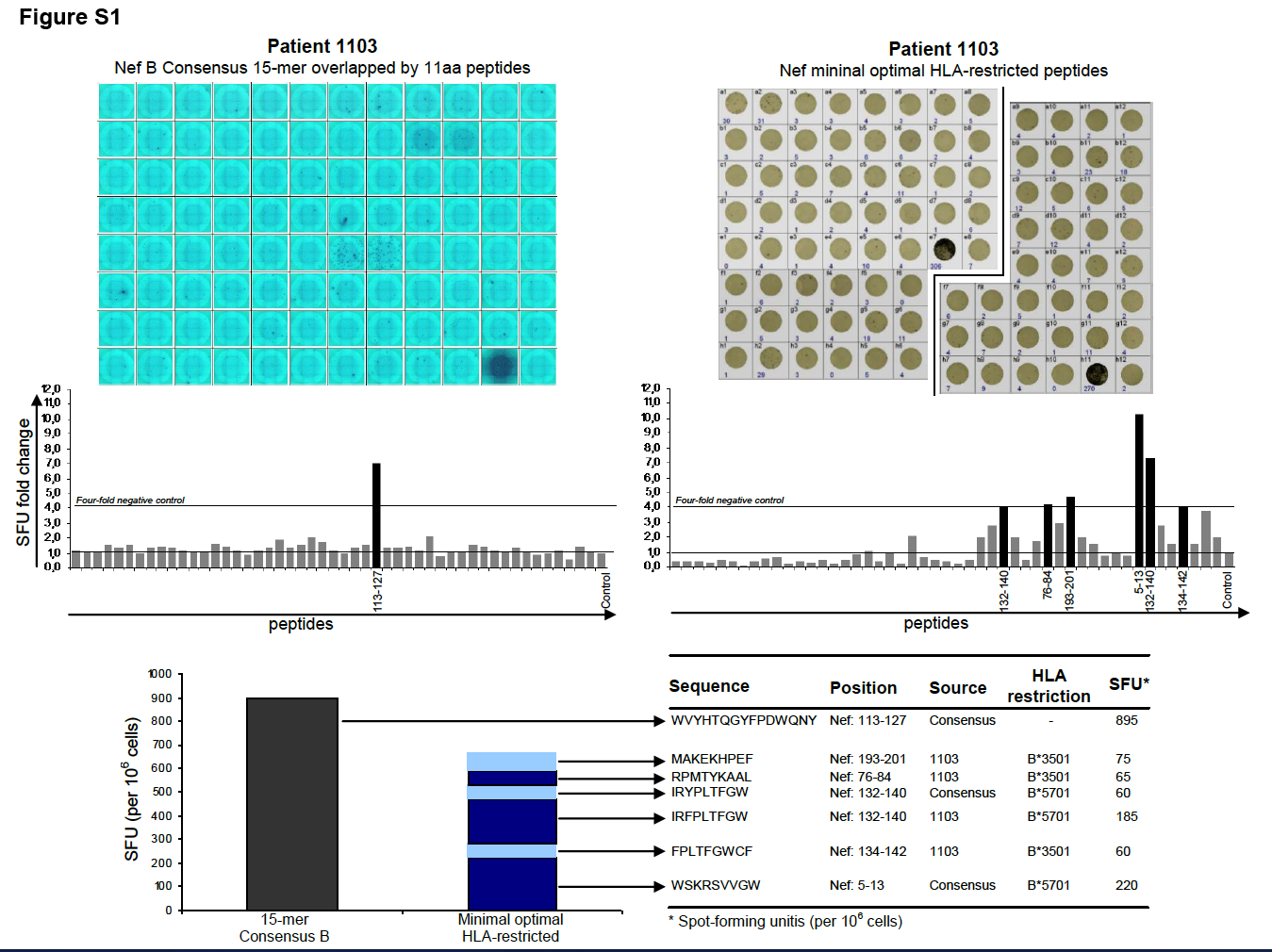

Supplement: Figure S1 — ELISPOT assays using 15mer consensus B peptides and HLA-restricted minimal optimal peptides. ELISPOT assays were done as described in the Methods section using either Vif and Nef 15mer consensus B peptides or Vif and Nef HLA-restricted minimal optimal peptides. For both assays, a concentration of 10 µg/mL for each peptide tested separately was used. A response was considered positive if the number of SFU exceeded 55 SFU per 106 cells and was at least four-times the level of the wells with no peptide. (4.11 MB TIF) [file pone.0011436.s003.tif]

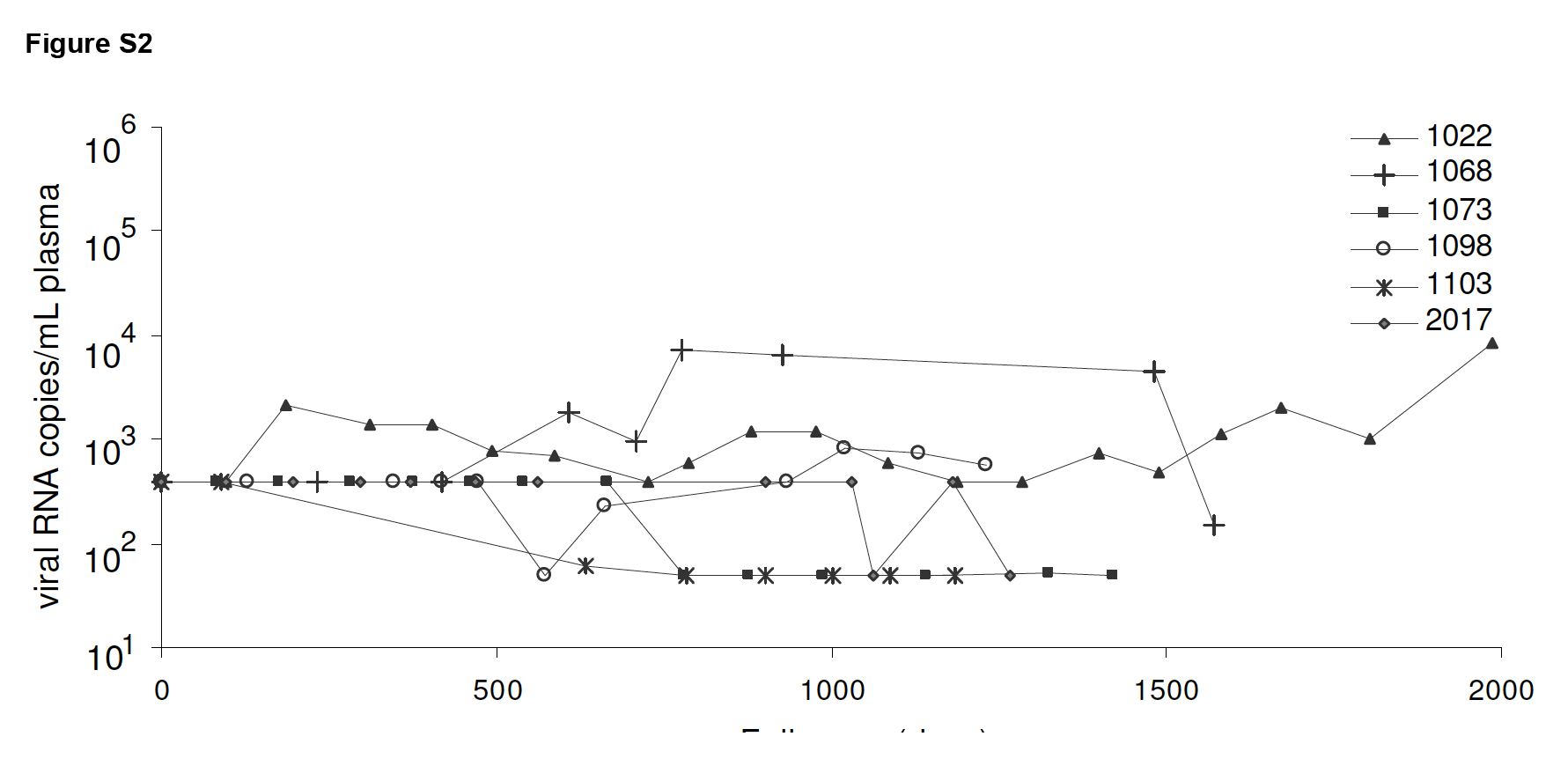

Supplement: Figure S2 — Viral load in the controller patients. Quantifications of viral load were performed by RT-PCR or branched DNA assays. The graph shows the values using a log scale. (6.22 MB TIF) [file pone.0011436.s004.tif]
